# *Staphylococcus aureus* Small RNAs Possess Dephospho-CoA 5′-Caps, but No CoAlation Marks

**DOI:** 10.3390/ncrna8040046

**Published:** 2022-06-28

**Authors:** Christian Löcherer, Nadja Bühler, Pascal Lafrenz, Andres Jäschke

**Affiliations:** Institute of Pharmacy and Molecular Biotechnology, Heidelberg University, Im Neuenheimer Feld 364, 69120 Heidelberg, Germany; c.loecherer@gmx.de (C.L.); nadjabuehler.98@gmx.de (N.B.); pascal.lafrenz@gmail.com (P.L.)

**Keywords:** CoA, CoA disulfide reductase, CoAlation, 3′-dephospho-CoA, dpCoA-RNA, RNA modification, APM, *S. aureus*

## Abstract

Novel features of coenzyme A (CoA) and its precursor, 3′-dephospho-CoA (dpCoA), recently became evident. dpCoA was found to attach to 5′-ends of small ribonucleic acids (dpCoA-RNAs) in two bacterial species (*Escherichia coli* and *Streptomyces venezuelae*). Furthermore, CoA serves, in addition to its well-established coenzymatic roles, as a ubiquitous posttranslational protein modification (‘CoAlation’), thought to prevent the irreversible oxidation of cysteines. Here, we first identified and quantified dpCoA-RNAs in the small RNA fraction of the human pathogen *Staphylococcus aureus*, using a newly developed enzymatic assay. We found that the amount of dpCoA caps was similar to that of the other two bacteria. We furthermore tested the hypothesis that, in the environment of a cell, the free thiol of the dpCoA-RNAs, as well as other sulfur-containing RNA modifications, may be oxidized by disulfide bond formation, e.g., with CoA. While we could not find evidence for such an ‘RNA CoAlation’, we observed that CoA disulfide reductase, the enzyme responsible for reducing CoA homodisulfides in *S. aureus*, did efficiently reduce several synthetic dpCoA-RNA disulfides to dpCoA-RNAs in vitro. This activity may imply a role in reversing RNA CoAlation.

## 1. Introduction

Redox homeostasis is an important prerequisite of life. To cope with harmful oxidative stress, nature takes advantage of the high reversibility of thiol-disulfide redox pairs. Most organisms utilize glutathione (GSH) as their principal antioxidative thiol [1]. Oxidation of GSH to the respective homodisulfide traps reactive oxygen species (ROS). A specialized enzyme, glutathione reductase, regenerates the free thiol form afterward [2]. Many organisms use additional thiol compounds, some even instead of GSH [1]. One example is *Staphylococcus aureus* (*S. aureus*). While GSH is completely absent, *S. aureus* utilizes coenzyme A (CoA) and bacillithiol as respective thiol-disulfide pairs [3,4,5,6]. CoA forms homodisulfides and mixed disulfides in response to ROS. Reversible disulfide formation between CoA and protein cysteines, also referred to as ‘protein CoAlation’, has been suggested to prevent protein damage by irreversible oxidation, although it is currently not known whether this process is chemically driven or enzymatically catalyzed [7,8]. Analogous to glutathione reductase, coenzyme A disulfide reductase (CoADR) assures a sufficient pool of reduced thiol species [9,10,11,12,13,14]. *S. aureus* CoADR is a flavoprotein disulfide reductase that utilizes NADPH to reduce CoA homodisulfides, but also several other low-molecular-weight disulfides [9]. One molecule of phosphopantetheine (PPant), a substructure of CoA, is the crucial binding motif [9,15].

In 2009, two studies discovered that 3′-dephospho-CoA (dpCoA) and nicotinamide adenine dinucleotide (NAD) can covalently attach to 5′-ends of ribonucleic acids (RNAs) [16,17]. These modified RNAs, termed dpCoA-RNAs and NAD-RNAs, were identified in the two bacterial species *Escherichia coli* (*E. coli*) and *Streptomyces venezuelae* (*S. venezuelae*), and consisted of less than 200 nucleotides (nt). NAD and dpCoA serve as non-canonical 5′-cap structures that are incorporated by numerous RNA polymerases (RNAPs) during transcription initiation [18,19,20,21]. NAD and dpCoA caps possess their own degradation pathways, thus critically influencing the fate of the associated RNAs [22,23,24,25,26,27]. NAD-RNAs have meanwhile been detected in various prokaryotes, eukaryotes, and also in RNA viruses [24,28,29,30,31,32,33]. Evidence for dpCoA-RNAs, on the other hand, remained restricted to two bacterial species [34].

In this manuscript, we identify and quantify dpCoA caps in the firmicute *S. aureus,* an organism with high concentrations of CoA and dpCoA [35]. We furthermore test the hypothesis that CoAlation is not restricted to proteins in this organism, but may also occur at thio-modifications in RNA, either at 5′-terminal dpCoA caps or at internal positions.

## 2. Results

### 2.1. Synthesis and Purification of dpCoA-RNA

**Figure 1:**
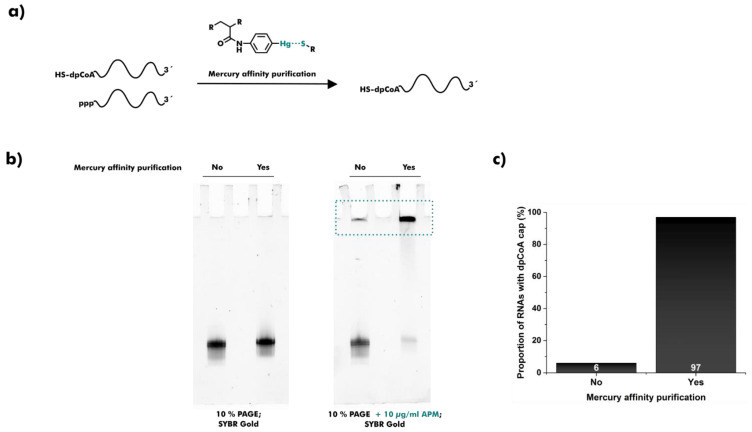
Mercury affinity purification of dpCoA-RNA. (**a**) Schematic overview of the purification principle. dpCoA-RNA is retarded by the interaction between its thiol and mercury (II) of co-polymerized 4-(acrylamido)phenylmercuric chloride (APM) during gel electrophoresis. (**b**) In vitro transcribed dpCoA-RNA was purified using 10% PAGE or a combination of 10% PAGE and 10% APM-PAGE. Both purification steps were analyzed using 10% PAGE (left gel) or 10% APM-PAGE (right gel) and SYBR Gold staining. The retarded dpCoA-RNA fraction is highlighted with a cyan frame. (**c**) Proportion of dpCoA-RNA before and after mercury affinity purification.

To develop a method for dpCoA cap quantification, we first synthesized a 107 nt-long model of dpCoA-RNA in vitro. T7 RNA polymerase was previously shown to initiate transcription partially with dpCoA instead of ATP if the ϕ2.5 T7 promoter and a high dpCoA to ATP ratio were used [18]. The resulting mixture of dpCoA-RNA and excessive 5′-triphosphate RNA (ppp-RNA) could not be resolved using standard gel electrophoresis. However, several affinity-based strategies have already been successfully applied to separate RNAs of equal length but different chemical composition. RNAs with 7-methylguanosine or NAD caps were efficiently retarded during polyacrylamide gel electrophoresis (PAGE) if 3-(acrylamido)phenylboronic acid (APB) was present [36,37]. Similarly, 4-(acrylamido)phenylmercuric chloride (APM) was used to retard and purify sulfur-containing transfer RNAs (tRNAs) and phosphorothioate-RNAs during gel electrophoresis [38]. We adapted the principle of mercury affinity electrophoresis (APM-PAGE) for the analysis and purification of dpCoA-RNA (Figure 1a). Gel elution in the presence of dithiothreitol (DTT) yielded dpCoA-RNA with a purity of 97% (Figure 1b,c).

### 2.2. Detection and Quantification of CoA and dpCoA-RNA

**Figure 2:**
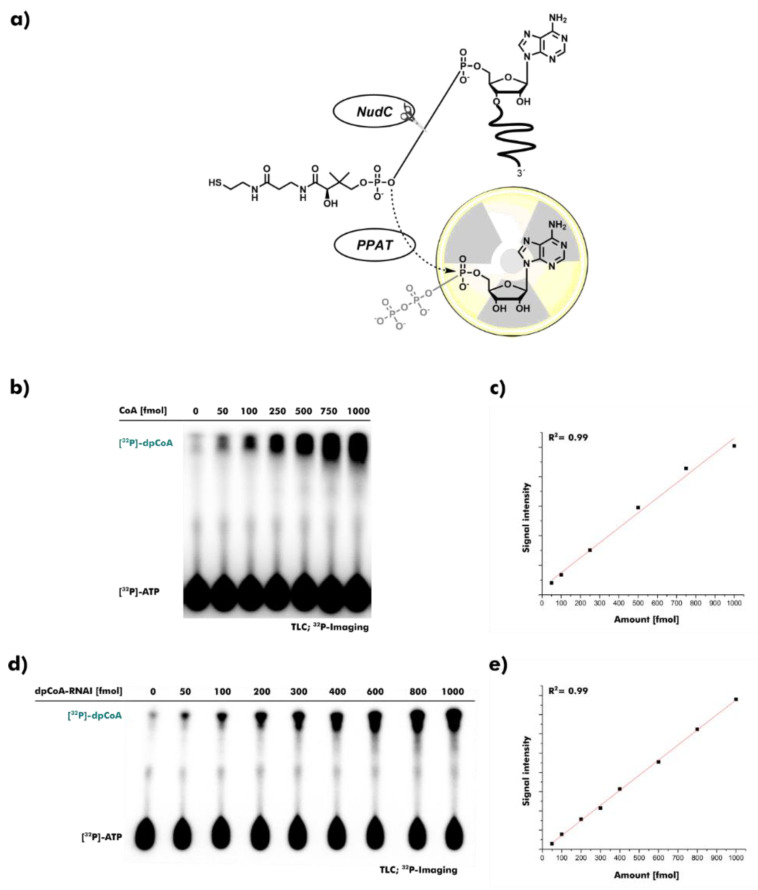
Assay development for the quantification of CoA and dpCoA-RNA. (**a**) Schematic overview of the assay principle. CoA or dpCoA-RNA is hydrolyzed to uniform phosphopantetheine (PPant) by *E. coli* nudix hydrolase NudC. PPant is subsequently converted to [α-^32^P]-dpCoA by *E. coli* phosphopantetheine adenylyltransferase (PPAT) in the presence of [α-^32^P]-ATP. (**b**) The [α-^32^P]-dpCoA signal for 50–1000 fmol CoA is separated using thin-layer chromatography (TLC) and visualized using ^32^P-imaging. (**c**) The linear correlation between the amount of CoA and [α-^32^P]-dpCoA signal is depicted. (**d**) The [α-^32^P]-dpCoA signal for 50–1000 fmol dpCoA-RNA is separated using TLC and visualized using ^32^P-imaging. (**e**) The linear correlation between the amount of dpCoA-RNA and [α-^32^P]-dpCoA signal is depicted.

We developed a bienzymatic assay for the sensitive detection of CoA and dpCoA-RNA (Figure 2a). The *E. coli* nudix hydrolase NudC is quite a promiscuous pyrophosphatase with activity towards NAD-RNA and dpCoA-RNA as well as their low-molecular-weight counterparts NAD and CoA [22,39,40]. NudC was used to transform CoA or dpCoA-RNA into uniform phosphopantetheine (PPant). Secondly, the assay included the CoA biosynthetic enzyme *E. coli* phosphopantetheine adenylyltransferase (PPAT), which catalyzes the conversion of PPant to dpCoA under consumption of adenosine triphosphate (ATP) [41]. [α-^32^P]-ATP was used to produce ^32^P-labeled dpCoA. The conversion of [α-^32^P]-ATP to [^32^P]-dpCoA was visualized after separation using thin-layer chromatography (TLC). CoA and dpCoA-RNA were reliably quantified in the range from 50 to 1000 femtomol (fmol) (Figure 2b–e). Acetyl-CoA was not detected by the assay, suggesting specificity for non-thioesterified CoA and dpCoA-RNA (Figure S1). The established enzymatic assay enables the analysis of multiple samples in parallel. The sensitivity is comparable to assays based on liquid chromatography-mass spectrometry, the current gold standard for the detection of CoA and dpCoA-RNAs [28,42,43].

### 2.3. dpCoA Capping and CoAlation in S. aureus Small RNA Isolates

**Figure 3:**
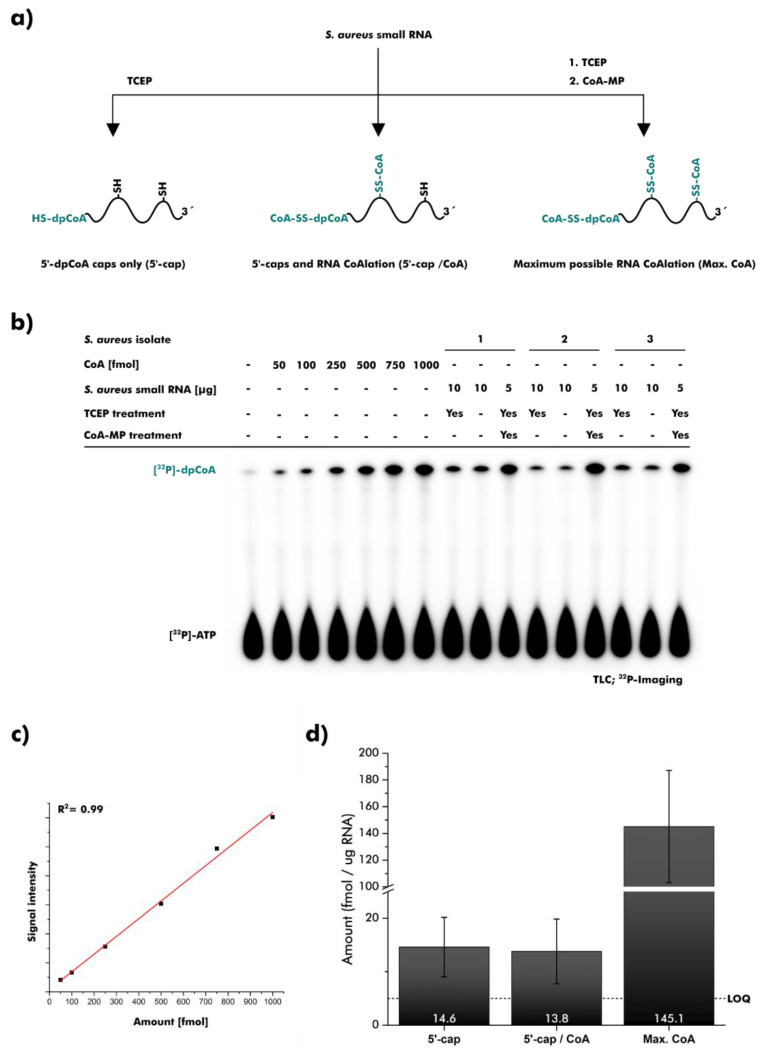
Quantification of dpCoA-RNAs and RNA CoAlation in *S. aureus*, grown in the absence of oxidative stress. (**a**) Schematic overview. Three different procedures were applied to small RNA isolates prior to quantification: treatment with tris(2-carboxyethyl)phosphine (TCEP), no pre-treatment, or treatment with TCEP and 2-mercaptopyridine-activated CoA (CoA-MP). (**b**) dpCoA caps and CoAlation events were analyzed after separation using thin-layer chromatography (TLC). Signals were visualized using ^32^P-imaging. [^32^P]-dpCoA signals were quantified. (**c**) CoA was used to create a calibration line for 50–1000 fmol CoA and dpCoA-RNA. (**d**) The bar chart illustrates the amount of dpCoA-RNAs and CoA in one microgram small RNA of *S. aureus*. 5′-dpCoA caps (5′-cap) were quantified. A combination of 5′-dpCoA caps and in vivo CoAlation events (5′-cap/CoA) was quantified. Potential CoAlation sites (Max. CoA) in *S. aureus* small RNA were quantified. Error bars represent the standard deviation (n = 3). The limit of quantification (LOQ) is 50 fmol.

We applied the assay to RNA isolated from the bacillithiol-deficient strain *S. aureus ATCC25923* [3]. The RNA fraction smaller than ~200 nucleotides (nt) was isolated and processed in three different ways (i–iii) (Figure 3a): (i) RNA samples were treated with the reducing agent tris(2-carboxyethyl)phosphine (TCEP) to remove CoA and its precursors were bound to RNA via disulfide bridges, allowing the exclusive detection of 5′-dpCoA caps. (ii) RNA samples were left untreated to detect dpCoA capping and in vivo RNA CoAlation. (iii) RNA samples were treated with TCEP followed by a large excess of 2-mercaptopyridine-activated CoA derivative (CoA-MP) to convert all reactive sulfur modifications to CoA disulfides [44,45]. This procedure should capture theoretically possible CoAlation sites. All samples were purified using 10% PAGE and subjected to quantification (Figure 3b). A calibration line was created with CoA (Figure 3c). For all samples, [^32^P]-dpCoA signals were consistently detected above the quantification limit (Figure 3d).

We detected 14.6 fmol dpCoA caps in 1 µg RNA, which is comparable to the amounts previously reported for *E. coli* (8 fmol/µg) and *S. venezuelae* (13 fmol/µg) [16]. No significant difference was observed when the treatment with TCEP was omitted, suggesting a lack of ‘RNA CoAlation’ (Figure 3d).

Pre-treatment with TCEP and CoA-MP resulted in a [^32^P]-dpCoA signal corresponding to 145.1 fmol/µg RNA (Figure 3d). Thus, dpCoA caps account for approximately 10% of CoAlation sites in *S. aureus* small RNA. Nucleobases with thioketone structure presumably represent another major portion, since these modifications are ubiquitous in tRNA and readily undergo disulfide formation [44].

We repeated the experiment with *S. aureus* cultures that were exposed to oxidative stress in the form of the oxidant diamide. Subsequent quantification of dpCoA caps and RNA CoAlation lead to almost identical results as for unstressed cultures (Figure S2a–c). Growth in the presence of diamide has been reported to dramatically increase protein CoAlation in various organisms, including *S. aureus* [8]. We questioned where the discrepancy between substantial protein CoAlation and absent RNA CoAlation might come from. A distinctive feature of *S. aureus* and other firmicutes is the presence of a specialized enzyme for the reduction of disulfides of CoA, namely CoA disulfide reductase (CoADR). This enzyme was recently proposed to be involved in the reversal of protein CoAlation [45].

### 2.4. Reduction of dpCoA-RNA Disulfides by S. aureus CoADR

We aimed to determine whether *S. aureus* CoADR can reverse 5′-CoAlation of dpCoA-RNAs in vitro. 5′-CoAlated dpCoA-RNA (CoA-SS-dpCoA-RNA) structurally resembles the natural substrate of CoADR except for a single-sided 3′-extension. We developed a strategy for the synthesis of CoA-SS-dpCoA-RNA, but also other dpCoA-RNA disulfides: Low-molecular-weight thiol compounds were first transformed into 2-mercaptopyridine-activated disulfides and then incubated with in vitro transcribed dpCoA-RNA to yield dpCoA-RNA disulfides (Figure S3a) [46,47]. The procedure was validated by the synthesis and analysis of ^32^P-body-labeled CoA-SS-dpCoA-RNA and dpCoA-SS-dpCoA-RNA (Figure S3b–d). APM-PAGE revealed the quantitative conversion of the substrate dpCoA-RNA after incubation with CoA-MP or dpCoA-MP (Figure S3c). The reaction products CoA-SS-dpCoA-RNA and dpCoA-SS-dpCoA-RNA could be differentiated using APB-PAGE because only the latter molecule contained an additional cis-diol in the cap structure (Figure S3d). The same strategy was then applied to synthesize non-^32^P-labeled dpCoA-RNA disulfides with CoA, dpCoA, and GSH (G-SS-dpCoA-RNA).

**Figure 4:**
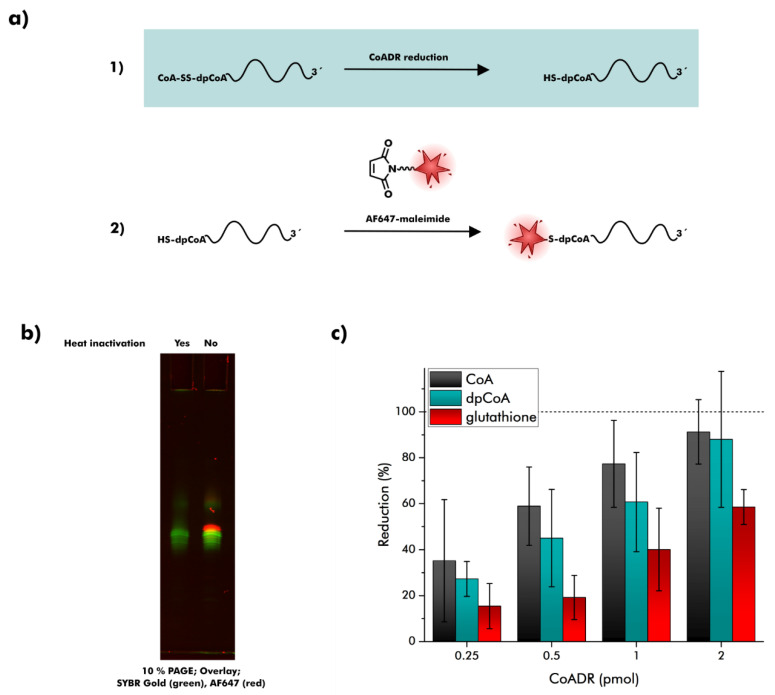
Reduction of dpCoA-RNA disulfides by *S. aureus* CoADR. (**a**) Schematic overview. (1) dpCoA-RNA disulfides were treated with *S. aureus* CoADR. (2) Originating dpCoA-RNAs were visualized with Alexa Fluor 647-maleimide (AF647-maleimide). (**b**) CoA-SS-dpCoA-RNA was incubated with either heat-inactivated or active *S. aureus* CoADR, then treated with AF647-maleimide. RNA was stained with SYBR Gold after 10% PAGE. AF647 signals (red) and SYBR Gold signals (green) were superimposed. (**c**) dpCoA-RNA disulfides with CoA, dpCoA and glutathione were incubated with defined concentrations of *S. aureus* CoADR or TCEP for 1 h, then treated with AF647-maleimide. AF647 signals were quantified after 10% PAGE. Gel segments with AF647 signals are shown in the supporting information (Figure S6). AF647 signal intensities were normalized to TCEP treatment (100% reduction). Error bars represent the standard deviation (n = 3).

We used a fluorescence-based approach to evaluate the substrate spectrum of *S. aureus* CoADR at the RNA level (Figure 4a). The RNA body was stained with SYBR Gold dye. Reduced dpCoA caps were additionally conjugated to Alexa Fluor 647-maleimide (AF647-maleimide). The Michael reaction between the thiol of dpCoA-RNA and AF647-maleimide exhibited excellent sensitivity, allowing detection of as little as 5 fmol of dpCoA-RNA, whereas 100 fmol of ppp-RNA produced no signal at all (Figure S4a–c). When we incubated CoA-SS-dpCoA-RNA with *S. aureus* CoADR, an AF647 signal appeared that co-localized with the SYBR Gold signal (Figure 4b), indicating the formation of dpCoA-RNA. This AF647 signal was absent after incubation with heat-inactivated *S. aureus* CoADR, suggesting an enzymatically driven reaction between the enzyme and the dpCoA-RNA disulfide. Remarkably, comparable results were obtained when dpCoA-SS-dpCoA RNA and G-SS-dpCoA RNA were treated with *S. aureus* CoADR, indicating a high binding affinity of the enzyme to the dpCoA-RNA moiety (Figure S5).

Kinetic parameters for the reduction of CoA homodisulfide, dpCoA homodisulfide, and CoA-GSH mixed disulfide by *S. aureus* CoADR have already been published [9]. CoA homodisulfide proved to be the preferred substrate, followed by dpCoA homodisulfide and CoA-GSH mixed disulfide. We assessed whether similar substrate preferences exist at the RNA level. CoA-SS-dpCoA-RNA, dpCoA-SS-dpCoA-RNA, and G-SS-dpCoA-RNA were incubated with increasing amounts of *S. aureus* CoADR (Figure 4c). Indeed, activity toward dpCoA-RNA disulfide substrates followed the same trend: reduction by *S. aureus* CoADR was highest for CoA-SS-dpCoA-RNA, intermediate for dpCoA-SS-dpCoA-RNA, and lowest for G-SS-dpCoA-RNA (Figure 4c and Figure S6).

## 3. Discussion

We developed a sensitive quantification method for CoA and dpCoA-RNAs and applied it to the small RNA fraction of *S. aureus*. We detected 11.2–14.6 fmol dpCoA caps per microgram of RNA. These values correspond to 0.03–0.04% dpCoA capping, assuming an average length of 85 nt (27.5 kDa) of the small RNA fraction. dpCoA caps are slightly less abundant than NAD caps (25.25 fmol/µg total RNA) [33]. *S. aureus* is the third bacterium after *E. coli* and *S. venezuelae* in which dpCoA-RNAs have been identified [16]. The elucidation of the identity, structure, function, and localization of dpCoA-capped RNAs, however, urgently requires a dpCoA-specific RNA enrichment and sequencing protocol, which does not exist to date. RNA capping with dpCoA may be a widespread phenomenon, as NAD-RNAs have also been detected in other domains of life [24,28,30,32]. Biosynthetically, both NAD and dpCoA serve as non-canonical 5′-cap structures that are incorporated by RNA polymerases during transcription initiation in a promoter-dependent manner [18,19,20,21]. We therefore expect, such as in the case of NAD-RNAs, specific RNA species to be dpCoA-capped.

ROS-promoted CoAlation was recently described for proteins [7,8]. We speculated on an equivalent RNA CoAlation, and quantified potential CoAlation sites and actual CoAlation events in isolated *S. aureus* small RNA. dpCoA caps were found to account for approximately 10% of the potential CoAlation sites. Strikingly, CoAlation events were completely absent in vivo, even after the application of oxidative stress during bacterial growth. This could be due to disulfide formation with thiol compounds other than CoA or due to active maintenance of the reduced state.

Inspired by a recent proposal on protein deCoAlation [45], we suspected *S. aureus* CoADR to play a role in the prevention of 5′-CoAlation. Intriguingly, the enzyme restored dpCoA-RNAs not only after CoAlation but also after disulfide formation with dpCoA and GSH in vitro. Whether *S. aureus* CoADR removes CoAlation marks in vivo can currently only be speculated.

## 4. Materials and Methods

### 4.1. General Procedures

All reagents were purchased from Sigma Aldrich unless otherwise stated. Chemicals were generally of reagent grade or ACS grade. Solvents were generally of HPLC grade or molecular biology grade. Reagents were generally of molecular biology grade. Ultrapure water was obtained from a Milli-Q Advantage A10 Water Purification System (Merck Millipore). All kits and enzymatic reactions were performed according to manufacturer instructions unless otherwise stated.

### 4.2. Synthesis of 4-(Acrylamido)Phenylmercuric Chloride (APM)

APM was mainly synthesized as described previously [48]. Under argon atmosphere, 497 mg 4-aminophenylmercuric acetate (1.41 mmol; 1 equivalent) was dissolved in 4 mL dry DMF and cooled to 0 °C. An amount of 145 µL acrylic acid (1.5 equivalents) and 698 mg 1-ethyl-3-(3-dimethylaminopropyl) carbodiimide (3.2 equivalents) was added and stirred at room temperature for 20 h. The product was precipitated by the addition of 10 mL deionized water. The product was washed three times with 10 mL deionized water and lyophilized. A total of 489 mg of product (1.28 mmol, 91% yield) was obtained. Product identity was confirmed using nuclear magnetic resonance (NMR) spectroscopy, performed on a Mercury plus 300 MHz spectrometer (Varian, Palo Alto, CA, USA).

^1^H NMR (300 MHz, DMSO-*d*_6_) δ 7.61 (d, *J* = 8.1 Hz, 2H), 7.40 (d, *J* = 8.2 Hz, 2H), 6.44 (dd, *J* = 17.0, 10.1 Hz, 1H), 6.24 (dd, *J* = 17.0, 2.2 Hz, 1H), 5.74 (dd, *J* = 10.0, 2.2 Hz, 1H).

### 4.3. Synthesis of 3-(Acrylamido)Phenylboronic Acid (APB)

APB was mainly synthesized as described previously [49]. An amount of 25 g 3-aminophenylboronic acid hemisulfate (134 mmol, 1 equivalent) was dissolved in 600mL deionized water and cooled to 0 °C. A total of 22.6 g sodium bicarbonate was added in portions under rigorous stirring. The solution was transferred at room temperature. An amount of 17 mL acryloyl chloride (210 mmol; 1.6 equivalents) was added dropwise under stirring over a period of 60 min. The reaction mixture was left stirring at room temperature for 30 min, then cooled to −20 °C. The precipitate was washed once with 100 mL ice-cold deionized water. The filter cake was dissolved in 250 mL ethyl acetate and dried over excessive anhydrous magnesium sulfate. Crude product was obtained after filtration and evaporation of ethyl acetate. Recrystallization was conducted in 150–180 mL deionized water at 95 °C. The hot solution was filtrated and slowly cooled to 0 °C. The precipitate was dried over anhydrous calcium chloride in vacuo. A total of 14.88 g of product (78 mmol, 58% yield) was obtained. Product identity was confirmed using NMR spectroscopy, performed on a Mercury plus 300 MHz spectrometer (Varian).

^1^H NMR (300 MHz, DMSO-*d*_6_) δ 7.88 (d, *J* = 2.2 Hz, 1H), 7.81 (d, *J* = 8.1 Hz, 1H), 7.49 (d, *J* = 7.2 Hz, 1H), 7.28 (t, *J* = 7.7 Hz, 1H), 6.45 (dd, *J* = 16.9, 10.1 Hz, 1H), 6.24 (dd, *J* = 17.1, 2.2 Hz, 1H), 5.73 (dd, *J* = 10.0, 2.2 Hz, 1H).

### 4.4. Synthesis of 2-Mercaptopyridine-Activated Disulfides

2-mercaptopyridine-activated disulfides of CoA (CoA-MP), dpCoA (dpCoA-MP) and glutathione (GSH-MP) were synthesized according to a general strategy: 20 µmol of the respective thiol compound was dissolved in 13 µL 100 mM triethylammonium acetate pH 7 (TEAA). An amount of 100 µmol Aldrithiol-2 was dissolved in 156 µL DMF. Both solutions were mixed and incubated at room temperature for 1 h. The reaction was quenched with 3.12 mL ultrapure water and filtrated through a 10 kDa Amicon Ultra centrifugal filter. The product was purified using reversed-phase high-performance liquid chromatography. The HPLC modules of Agilent 1100 series (Agilent Technologies, Waldbronn, Germany) were equipped with a Luna C18 (250 mm × 15 mm; 5 µM, 100 Å, Phenomenex Aschaffenburg, Germany). The solvent system consisted of 100 mM TEAA pH 7 and acetonitrile (MeCN). Elution was performed in a linear gradient starting at 1.6% MeCN. The product peak was identified at 260 nm and lyophilized. The product was dissolved in ultrapure water and the concentration was determined in the presence of excessive 2-mercaptoethanol. The amount of liberated 2-mercaptopyridine (ε_343 nm_ = 7600 L × mol^−1^ × cm^−1^) was detected at 343 nm on Nanodrop One (Thermo Fisher Scientific, Karlsruhe, Germany) [50]. The yields were 90% for CoA-MP, 34% for dpCoA-MP, and 8% for GSH-MP. The product identity was confirmed using mass spectrometry in negative mode using electrospray ionization (ESI) in combination with time-of-flight (TOF) detection on a micrOTOF QII system (Bruker):

dpCoA-MP: [M-H]^−^ calculated for C_26_H_38_N_8_O_13_P_2_S_2_: 795.15, found: 795.2

CoA-MP: [M-H]^−^ calculated for C_26_H_39_N_8_O_16_P_3_S_2_: 875.1066, found: 875.1083.

GSH-MP: [M-H]^−^ calculated for C_15_H_20_N_4_O_6_S_2_: 415.0751, found: 415.0751.

### 4.5. Polyacrylamide Electrophoresis (PAGE)

All samples were mixed with one volume of formamide prior to loading. Polyacrylamide electrophoresis (PAGE) was always performed under denaturing conditions in the presence of 7.5 M urea and 10% acrylamide/bisacrylamide (19:1, Carl Roth, Karlsruhe, Germany). PAGE was performed in TBE buffer (100 mM Tris-borate pH 8.3, 2 mM ethylenediaminetetraacetic acid (EDTA)) at constant power setting. Products were visualized using different techniques. UV shadowing at 254 nm was used in preparative scale. Otherwise, RNAs were exposed to SYBR Gold (ThermoFisher Scientific, Karlsruhe, Germany, excitation at 473 nm) or phosphor storage screens (Amersham Biosciences, excitation at 635 nm) and visualized on a Typhoon FLA 9500 Biomolecular Imager (Cytiva). Signals were quantified with ImageQuant TL (Cytiva, Freiburg, Germany). Preparative workup was conducted as follows. Gel pieces were excised. RNAs were eluted in 300 mM sodium acetate pH 5.5 at 15 °C for 15 h, passed through a Maxi-Spin 25 mL nylon filter (Frisenette, Knebel, Denmark), and precipitated with 1 volume isopropanol. Nucleic acid pellets were dissolved in ultrapure water by default. Nucleic acid concentrations were determined using UV absorbance at 260 nm on Nanodrop One (ThermoFisher Scientific), unless otherwise stated.

### 4.6. Affinity Gel Electrophoresis (APM-PAGE and ABP-PAGE)

APM-PAGE and APB-PAGE were conducted as stated above with minor modifications. APB-PAGE was performed in TAE buffer (100 mM Tris-acetate, 5 mM EDTA, pH 8.0). For APM-PAGE, 1 mg/mL APM stock solution in DMF was added to the polyacrylamide mix to a final concentration of 10 µg/mL prior to polymerization. For APB-PAGE, solid APB was dissolved in the polyacrylamide mix at 65 °C for 5 min to a final concentration of 0.5% (*w*/*v*) prior to polymerization. Products were visualized and quantified as stated above. Elution from APM-containing gels was performed in the presence of 300 mM sodium acetate pH 5.5 and 100 mM dithiothreitol (DTT). APM-containing gels were disposed of separately as hazardous waste.

### 4.7. Synthesis of dpCoA-RNA

Synthesis of dpCoA-RNA was mainly performed as described previously [18]. Transcription was conducted in the presence of 2 mM dpCoA (Cayman Chemical, Ann Arbor, MI, USA), 1 mM ATP, 2 mM CTP, 2mM UTP, 2mM GTP, 40 mM Tris-HCl pH 8.0, 22 mM MgCl_2_, 1 mM spermidine, 0.01% Triton-X-100, 5% dimethyl sulfoxide (DMSO), 10 mM dithiothreitol (DTT), 12.5 ng/µL template, and 250 ng/µL T7 RNA polymerase at 37 °C for 4 h. An amount of 1.25 µM α-^32^P-CTP (3000 Ci/mmol, Hartmann Analytics, Braunschweig, Germany) was included, if visualization using ^32^P-imaging was desired. The 107 nucleotide (nt)-long *E. coli* RNAI sequence with ϕ2.5 T7 promoter was chosen as a double-stranded DNA template (Integrated DNA Technologies, Coralville, IA, USA) desalted).

RNA sequence (5′-3′):

ACAGUAUUUGGUAUCUGCGCUCUGCUGAAGCCAGUUACCUUCGGAAAAAGAGUUGGUAGCUCUUGAUCCGGCAAACAAACCACCGCUGGUAGCGGUGGUUUUUUUGU

Transcription products were purified using 10% PAGE followed by 10% APM-PAGE. The concentration was determined using the Qubit RNA high sensitivity assay on a Qubit 2.0 fluorometer (both ThermoFisher Scientific).

### 4.8. Synthesis of dpCoA-RNA Disulfides

The three dpCoA-RNA disulfides CoA-SS-dpCoA-RNAI, dpCoA-SS-dpCoA-RNAI, and G-SS-dpCoA-RNAI were synthesized according to a general strategy: 1 nmol dpCoA-RNA was incubated with 10 nmol CoA-MP, dpCoA-MP, or GSH-MP in 50 mM Tris HCl pH 8 at 25 °C and 300 rpm shaking for 1 h. The total volume was 10 µL. The reaction mixture was purified using 10% PAGE.

### 4.9. Bacterial Growth

*E. coli* colonies (for strain details, see following paragraphs) were grown on LB agar (Lennox, Carl Roth, Karlsruhe, Germany) supplemented with 30 µg/mL kanamycin (Carl Roth) at 37 °C. *E. coli* BL21(DE3) and *E. coli* DH5α (both ThermoFisher Scientific) liquid cultures were grown in LB medium (Lennox, Carl Roth) supplemented with 30 µg/mL kanamycin, except for transformations in which kanamycin was omitted. *E. coli* K-12 (DSMZ) liquid cultures were grown in LB medium (Lennox). *S. aureus* ATCC25923 colonies were a kind gift from Dennis Nurjadi (Department of Infectious Diseases, University Hospital Heidelberg, Heidelberg). *S. aureus* ATCC25923 liquid cultures were grown in LB medium (Lennox). All liquid cultures were grown in baffled Erlenmeyer flasks in a MaxQ 8000 shaker (ThermoFisher Scientific) at 37 °C and 165 rpm shaking.

### 4.10. Molecular Cloning of S. aureus Coenzyme A Disulfide Reductase

Genomic DNA of *S. aureus* ATCC25923 was isolated as follows. 1.5 mL of overnight culture was pelleted and washed with 500 µL TE buffer (30 mM Tris HCl, 1 mM EDTA) pH 8. The pellet was resuspended in 200 µL TE buffer pH 8 supplemented with 10 µg lysostaphin and incubated at 37 °C and 750 rpm shaking for 1 h. An amount of 15 µL of 20 mg/mL proteinase K was added and the mixture was incubated at 56 °C and 750 rpm shaking for 1 h. The mixture was heated to 95 °C for 15 min, then extracted twice with 1 volume of ROTI Aqua-P/C/I pH 7.5–8 (CarlRoth) and once with 3 volumes of diethyl ether. Residual organic layer was removed in vacuo. DNA was precipitated in the presence of 300 mM sodium acetate and 2.5 volumes of ethanol at −20 °C. The pellet was washed once with 1 mL 80% ethanol and dried in vacuo. The pellet was resuspended in TE buffer pH 8 and the concentration and purity were determined on Nanodrop One. Genomic DNA was used to amplify the cdr gene by polymerase chain reaction (PCR) using Q5 Hot Start High-Fidelity DNA Polymerase (New England BioLabs, Frankfurt, Germany) in a T-100 Thermal Cycler (Bio-Rad, Feldkirchen, Germany). The primers (Integrated DNA Technologies, desalted) included NcoI and XhoI restriction sites.

Forward primer: CGACCCATGGCGCCCAAAATAGTCGTAGTCGGAGCAGTCGCTGGCGG

Reverse primer: GGTGCTCGAGTTTAGCTTTGTAACCAATCATATTGATTAAATCTTTAGGGTGGC

The PCR reactions were purified with the QIAquick PCR Purification Kit (Qiagen, Hilden, Germany). Restriction digests of 2.5 µg PCR product and 2.5 µg pET28a vector were performed with FastDigest NcoI and FastDigest XhoI (both ThermoFisher Scientific, Karlsruhe, Germany). The digested cdr gene was purified with the QIAquick PCR Purification Kit (Qiagen). The digested vector was separated on 0.8% (m/V) agarose gel and purified with the QIAquick Gel Extraction Kit (Qiagen). An amount of 30 ng cdr insert was ligated into 30 ng vector by T4 DNA ligase (ThermoFisher Scientific) at 19 °C for 15 h, then incubated at 65 °C for 10 min. The mixture was transformed into *E. coli* DH5α (ThermoFisher Scientific). The transformation mixture was plated to obtain single colonies.

### 4.11. Protein Expression

*E. coli* nudix hydrolase NudC, *E. coli* phosphopantetheine adenylyltransferase (PPAT, coaD), and *S. aureus* CoA disulfide reductase (CoADR) were expressed in *E. coli* BL21(DE3). *E. coli* BL21(DE3) transformed with pET28a-NudC were a kind gift from Katharina Höfer (Max Planck Institute for Terrestrial Microbiology, Marburg). *E. coli* DH5α with pET28a-Ec.coaD (pESC106) were a gift from Tadhg Begley & Erick Strauss (Addgene plasmid # 50388). Plasmids encoding for PPAT or CoADR were isolated with the GeneJET Plasmid Miniprep Kit (ThermoFisher Scientific). Correct inserts were validated using Sanger sequencing (Microsynth SeqLab GmbH, Göttingen, Germany). Plasmids were transformed into *E. coli* BL21(DE3) (ThermoFisher Scientific) and plated to obtain single colonies.

1 L of bacterial culture was grown to an OD_600_ of 0.6–0.8. Expression of proteins with N-terminal 6x His-tags was induced by the addition of isopropyl β-D-1-thiogalactopyranoside (IPTG) to a final concentration of 1 mM. After 3 h, the bacteria were pelleted and washed with 15 mL Dulbecco’s phosphate buffer saline (PBS). The pellet was resuspended in FPLC buffer A (300 mM NaCl, 1 mM imidazole, 50 mM Tris HCl pH 8, and 5% (*v*/*v*) glycerol) and cells were lysed using sonication. Cell debris was pelleted using ultracentrifugation. The filtrated supernatant was subjected to fast protein liquid chromatography (FPLC). Samples were loaded onto a HisTrap HP 1 mL column (GE Healthcare) in an NGC chromatography system (BioRad). Elution was performed with a gradient from 3% to 100% FPLC buffer B (300 mM NaCl, 300 mM imidazole, 50 mM Tris HCl pH 8, and 5% (*v*/*v*) glycerol). Eluted fractions were analyzed using 12% SDS-PAGE and Coomassie staining. PageRuler Prestained Protein Ladder (ThermoFisher Scientific) was used to estimate the lengths of the protein bands. Pure protein fractions of correct size were combined, washed with storage buffer (300 mM NaCl, 50 mM Tris HCl pH 8, and 5% (*v*/*v*) glycerol) and concentrated using 10 kDa filtration. For CoADR, an additional size-exclusion purification on a Superdex 200 10/300 GL column (GE Healthcare) was conducted isocratically in storage buffer. Protein fractions were again analyzed using 10% SDS-PAGE and Coomassie staining. Pure protein fractions of correct size were combined and concentrated using 10 kDa filtration. Concentrations were determined using UV absorbance at 280 nm on Nanodrop One (ThermoFisher Scientific) and stored in 50% glycerol.

### 4.12. Small RNA Isolation

A total of 60 mL cultures of *S. aureus* ATCC25923 were grown to an OD_600_ of 0.7. If oxidative stress should be applied, diamide (TCI Chemicals) was added to a final concentration of 2 mM. This procedure was previously reported to increase protein CoAlation [8]. Bacteria were pelleted after 30 min incubation and resuspended in 1.4 mL TE buffer (30 mM Tris HCl, 1 mM EDTA) pH 8 supplemented with 40 mg/mL lysozyme and 40 µg/mL lysostaphin. The mixture was incubated at 37 °C and 150 rpm shaking for 1 h. The small RNA fraction smaller than 200 nt was isolated with 16 mL of RNAzol RT according to the manufacturer protocol. Obtained RNA samples in 50 mM Tris HCl pH 7.5 were divided into three equal aliquots. The first aliquot was treated with 10 nmol TCEP. The second aliquot was treated with buffer only. The third aliquot was treated with 10 nmol TCEP, purified using 10% PAGE, then treated with 58 nmol CoA-MP. The total volume was 50 µL each time. All reactions were incubated at 25 °C for 2 h. All samples were finally purified using 10% PAGE to a size range of ~50–200 nt.

### 4.13. Quantification of CoA and dpCoA-RNA

The assay was conducted in 0.2 mL tubes. Incubation steps were carried out in a thermal cycler (PTC-100, MJ Research) The sample of interest was treated with 50 pmol NudC in 1 mM DTT, 50 mM NaCl, 50 mM KCl, 10 mM MgCl_2_, 25 mM Tris HCl pH 7.5 at 37 °C for 3 h. The final reaction volume was 20 µL. The mixture was heated to 95 °C for 5 min and cooled to room temperature. An amount of 1 µL of 10 µM α^32^P-ATP (Hartmann Analytics) and 1 µL of 1 µM PPAT was added. The mixture was incubated at 37 °C for 3 h and analyzed using thin-layer chromatography (TLC) afterward. A total of 1 µL was spotted on Alugram Xtra SIL G UV_254_ plates (Macherey-Nagel) and separated in a solvent system of 1 M ammonium acetate pH 7 and ethanol (4:6). Phosphor storage screens (excitation at 635 nm) were visualized on Typhoon FLA 9500 Biomolecular Imager (Cytiva). Signals were quantified with ImageQuant TL (Cytiva). An amount of 50–1000 fmol of commercial CoA (Larodan, Solna, Sweden) and APM-PAGE-purified dpCoA-RNA, 1000 fmol of commercial acetyl-CoA, 10 µg of TCEP-treated and untreated *S. aureus* ATCC25923 small RNA, and 5 µg of CoA-MP-treated *S. aureus* ATCC25923 small RNA were quantified with this procedure.

### 4.14. S. aureus CoADR Reaction

Reaction conditions for *S. aureus* CoADR are orientated on a previous report [9]. If applicable, incubation at 95 °C for 10 min was used to inactivate *S. aureus* CoADR prior to the reaction. An amount of 1 pmol dpCoA-RNA disulfide was incubated with 2 nmol NADPH and varying concentrations of *S. aureus* CoADR in 50 mM Tris HCl pH 7.8 and 50 mM NaCl at 37 °C for 1 h. The total volume was 20 µL. The reactions were evaluated using fluorescent labeling of the product dpCoA-RNA.

### 4.15. Fluorescent Labeling of dpCoA-RNA

A total of 20 µL of RNA sample was incubated with 20 µL of aqueous 100 µM Alexa Fluor 647-maleimide (AF647-maleimide; Jena Bioscience, Jena, Germany) under light protection at 23 °C for 30 min. An amount of 20 µL of 3 M sodium acetate pH 5.5 and 140 µL ultrapure water was added. Protein and excessive dye were removed using threefold extraction with 200 µL ROTI Aqua Phenol pH 4.5–5 (Carl Roth). Residual phenol was extracted with 1 mL diethyl ether. RNA was precipitated with 2.5 volumes of ethanol. Samples were analyzed using 10% PAGE and stained with SYBR Gold (ThermoFisher Scientific) afterward. AF647 (excitation at 635 nm) and SYBR Gold (excitation at 473 nm) signals were visualized on Typhoon FLA 9500 Biomolecular Imager (Cytiva). Signals were quantified with ImageQuant TL (Cytiva).

## Data Availability

Not applicable.

## Supplementary Materials

The following are available online at https://www.mdpi.com/article/10.3390/ncrna8040046/s1, Figure S1: Selective quantification of non-thioesterified CoA. Figure S2: Quantification of dpCoA-RNA and RNA CoAlation in S. aureus, grown in the presence of diamide. Figure S3: Synthesis of dpCoA-RNA disulfides. Figure S4: Fluorescent labeling of dpCoA-RNA. Figure S5: Reduction of different dpCoA-RNA disulfides by S. aureus CoADR. Figure S6: Substrate preferences of S. aureus CoADR.
