# Supplementary material for: Staphylococcus aureus Small RNAs Possess Dephospho-CoA 5′-Caps, but No CoAlation Marks
Source: Noncoding RNA. 2022 Jun 28;8(4):46. doi: 10.3390/ncrna8040046 (PMC9326634; doi:10.3390/ncrna8040046)
Supplement: Supplementary file 1 [file ncrna-08-00046-s001.zip › ncrna-1792839-supplementary.pdf]

Supporting information

Staphylococcus aureus small RNAs possess dephospho-CoA 5′-caps, but no CoAlation marks.

Christian Löcherer [1], Nadja Bühler [1], Pascal Lafrenz [1] and Andres Jäschke [1,*]

[1] Institute of Pharmacy and Molecular Biotechnology, Heidelberg University, Im Neuenheimer Feld 364, Heidelberg, 69120, Germany; c.loecherer@gmx.de; nadjabuehler.98@gmx.de; pascal.lafrenz@gmail.com

* Correspondence: jaeschke@uni-hd.de; Tel.: +49-6221-544851

Supporting figures

Figure S1 Selective quantification of non-thioesterified CoA.

Figure S2 Quantification of dpCoA-RNA and RNA CoAlation in *S. aureus*, grown in the presence of diamide.

Figure S3 Synthesis of dpCoA-RNA disulfides.

Figure S4 Fluorescent labeling of dpCoA-RNA.

Figure S5 Reduction of different dpCoA-RNA disulfides by *S. aureus* CoADR.

Figure S6 Substrate preferences of *S. aureus* CoADR.

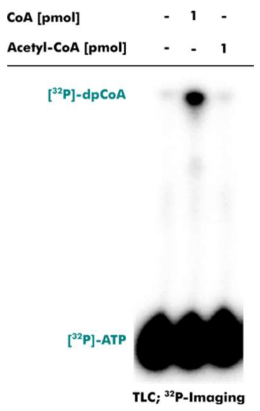

Figure S1. Selective quantification of non-thioesterified CoA. CoA and Acetyl-CoA were quantified with *E. coli* NudC, *E. coli* PPAT, and [α-^{32}P]-ATP. Arising [α-^{32}P]-dpCoA signals were separated by thin-layer chromatography (TLC) and visualized by ^{32}P-imaging.

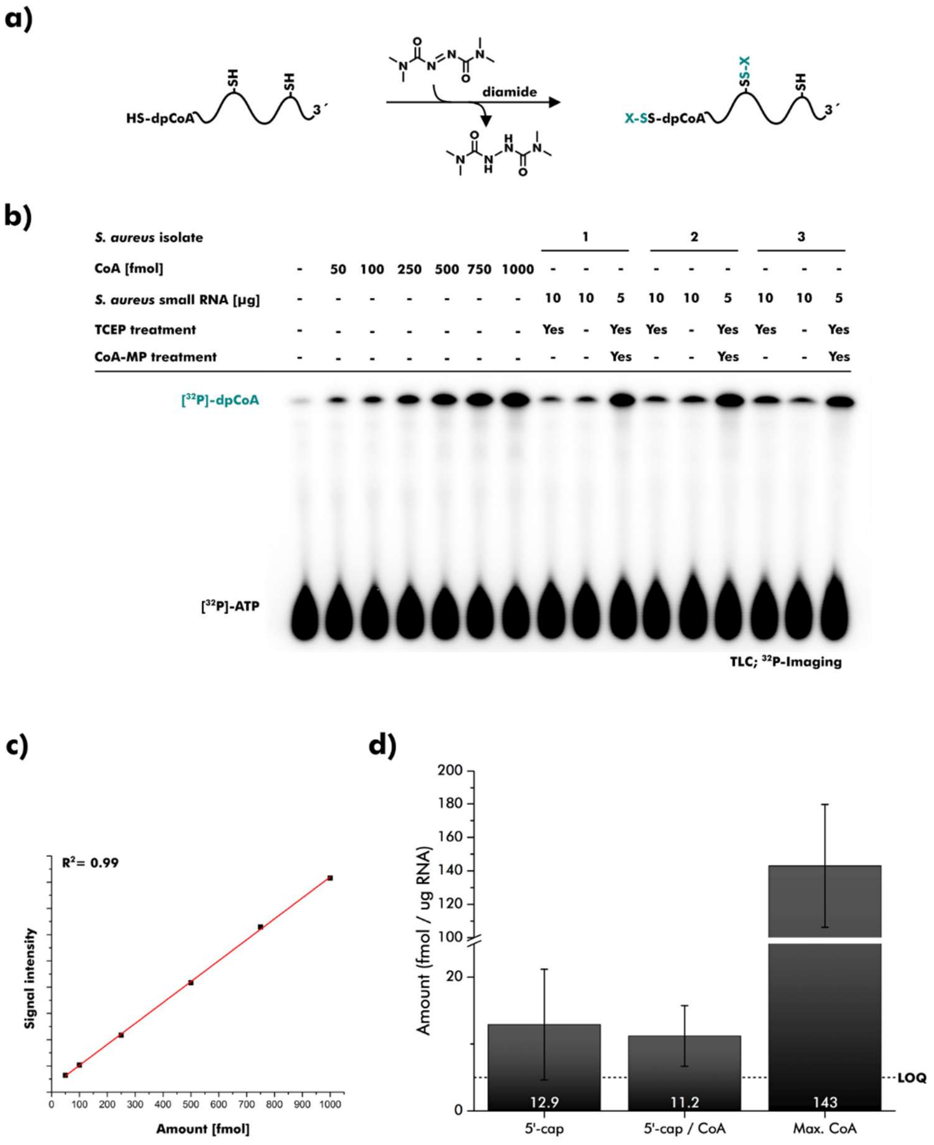

Figure S2 Quantification of dpCoA-RNA and RNA CoAlation in *S. aureus*, grown in the presence of diamide. **a)** Principle of increased in vivo RNA disulfide formation by the oxidant diamide. **b)** dpCoA caps and CoAlation events were analyzed after separation by thin-layer chromatography (TLC). Signals were visualized by ^{32}P-imaging. [^{32}P]-dpCoA signals were quantified. CoA-MP: 2-mercaptopyridine-activated CoA. **c)** CoA was used to create a calibration line for 50 – 1000 fmol CoA and dpCoA-RNA, respectively. **d)** The bar chart illustrates the amount of dpCoA-RNAs and CoA in one microgram small RNA of *S. aureus*. 5´-dpCoA caps (5´-cap) were quantified. A combination of 5´-dpCoA caps and *in vivo* CoAlation events (5´-cap/CoA) was quantified. Potential CoAlation sites (Max. CoA) in *S. aureus* small RNA were quantified. Error bars represent the standard deviation (n = 3). The limit of quantification (LOQ) is 50 fmol.

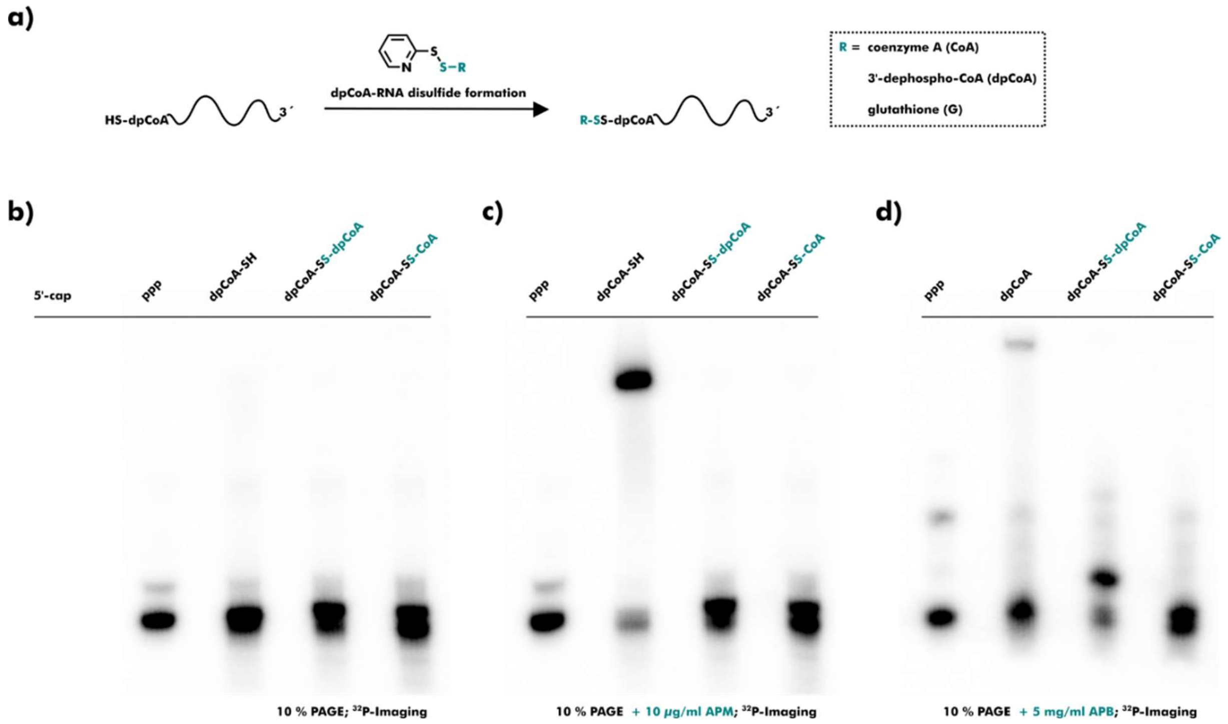

Figure S3 Synthesis of dpCoA-RNA disulfides. (**a**) dpCoA-RNA disulfides were produced by the reaction of ^{32}P-bodylabeled dpCoA-RNA with 2-mercaptopyridine-activated CoA and dpCoA. (**b - d**) The products CoA-SS-dpCoA-RNA and dpCoA-SS-dpCoA-RNA were analyzed by 10 % PAGE (b), 10 % APM-PAGE (c), and 10 % APB-PAGE (d). 5´-triphosphate-RNA (ppp-RNA) and the substrate dpCoA-RNA were analyzed simultaneously. Products were visualized by ^{32}P-imaging (excitation of phosphor storage screen at 635 nm).

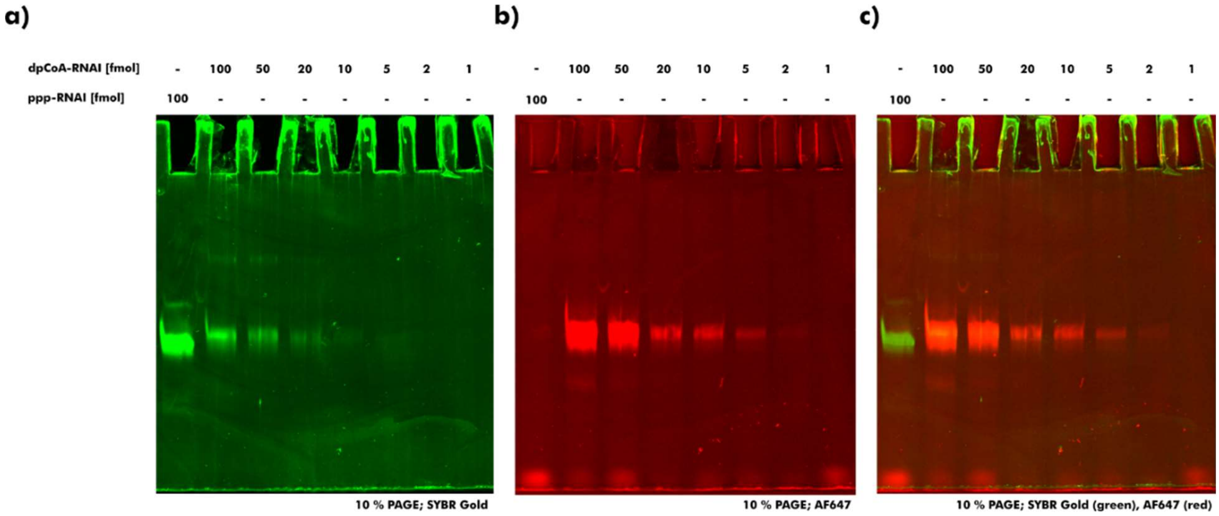

Figure S4 Fluorescent labeling of dpCoA-RNA. ppp-RNA and varying concentrations of dpCoA-RNA were incubated with Alexa Fluor 647-maleimide (AF647-maleimide). Products were and run on 10 % PAGE and stained with SYBR Gold. **a)** SYBR Gold visualization (excitation at 473 nm). **b)** AF647 visualization (excitation at 635 nm). **c)** SYBR Gold signals (green) and AF647 signals (red) were superimposed.

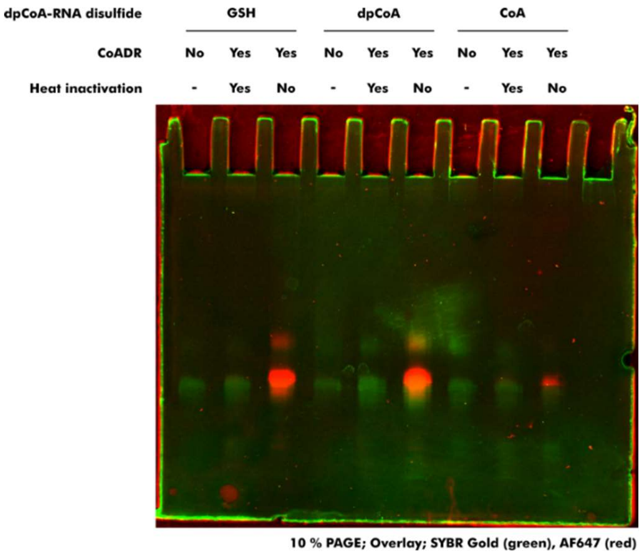

Figure S5 Reduction of different dpCoA-RNA disulfides by *S. aureus* CoADR. RNA were left untreated or incubated with either heat-inactivated or active *S. aureus* CoADR. Samples were incubated with AF647-maleimide and separated by 10 % PAGE. RNA were stained with SYBR Gold. AF647 signals (red; excitation at 635 nm) and SYBR Gold signals (green; excitation at 473 nm) were superimposed.

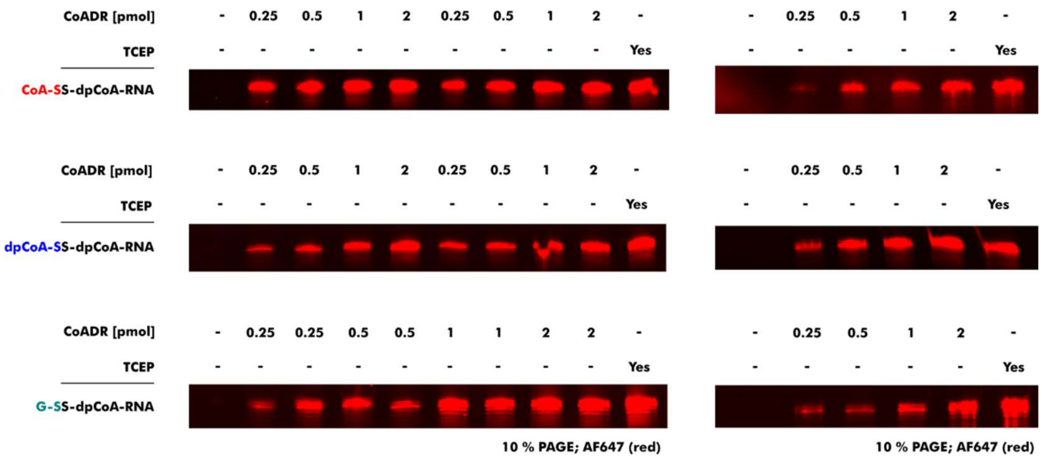

Figure S6 Substrate preferences of *S. aureus* CoADR. dpCoA-RNA disulfides with CoA (CoA-SS-dpCoA-RNA), dpCoA (dpCoA-SS-dpCoA-RNA) and glutathione (G-SS-dpCoA-RNA) were incubated with defined concentrations of *S. aureus* CoADR or TCEP for 1 h, then treated with AF647-maleimide. Gel segments with quantified AF647 signals
(excitation at 635 nm) are depicted.
